# Nonlinear phenotypic variation uncovers the emergence of heterosis in *Arabidopsis thaliana*

**DOI:** 10.1371/journal.pbio.3000214

**Published:** 2019-04-24

**Authors:** François Vasseur, Louise Fouqueau, Dominique de Vienne, Thibault Nidelet, Cyrille Violle, Detlef Weigel

**Affiliations:** 1 Max Planck Institute for Developmental Biology, Tübingen, Germany; 2 CEFE, CNRS, Univ Montpellier, Univ Paul Valéry Montpellier, EPHE, IRD, Montpellier, France; 3 Laboratoire d’Ecophysiologie des Plantes sous Stress Environnementaux (LEPSE), INRA, Montpellier SupAgro, UMR759, Montpellier, France; 4 GQE–Le Moulon, INRA, Univ Paris-Sud, CNRS, AgroParisTech, Univ Paris-Saclay, Gif-sur-Yvette, France; 5 SPO, INRA, Montpellier SupAgro, Univ Montpellier, Montpellier, France; Indiana University, UNITED STATES

## Abstract

Heterosis describes the phenotypic superiority of hybrids over their parents in traits related to agronomic performance and fitness. Understanding and predicting nonadditive inheritance such as heterosis is crucial for evolutionary biology as well as for plant and animal breeding. However, the physiological bases of heterosis remain debated. Moreover, empirical data in various species have shown that diverse genetic and molecular mechanisms are likely to explain heterosis, making it difficult to predict its emergence and amplitude from parental genotypes alone. In this study, we examined a model of physiological dominance initially proposed by Sewall Wright to explain the nonadditive inheritance of traits like metabolic fluxes at the cellular level. We evaluated Wright’s model for two fitness-related traits at the whole-plant level, growth rate and fruit number, using 450 hybrids derived from crosses among natural accessions of *A*. *thaliana*. We found that allometric relationships between traits constrain phenotypic variation in a nonlinear and similar manner in hybrids and accessions. These allometric relationships behave predictably, explaining up to 75% of heterosis amplitude, while genetic distance among parents at best explains 7%. Thus, our findings are consistent with Wright’s model of physiological dominance and suggest that the emergence of heterosis on plant performance is an intrinsic property of nonlinear relationships between traits. Furthermore, our study highlights the potential of a geometric approach of phenotypic relationships for predicting heterosis of major components of crop productivity and yield.

## Abstract 2

The phenotypic superiority of hybrids — “hybrid vigour” or heterosis — is often considered to be a biological mystery. Rooted on the mathematical modelling of trait variation, however, this study demonstrates that it can be explained simply with the non-linearity of phenotypic relationships.

## Author summary

Hybrids often grow faster and produce more offspring than their parents. This phenomenon, called hybrid vigour or heterosis, has been extensively exploited in agriculture. Even though several hypotheses have been proposed to explain the genetic basis of hybrid superiority, there is still no unifying model able to accurately predict the extent and amplitude of heterosis. Here, we tested a model based on physiological constraints to explain the emergence of heterosis in the model plant *Arabidopsis thaliana*. Rooted in the mathematical modelling of phenotypic relationships, our study demonstrates that heterosis can be explained simply with the nonlinearity of trait variation. Our model notably predicts the amplitude of heterosis of two performance traits: growth rate and fruit production. We expect that our model can help to understand the physiology of nonadditive inheritance in different species and that it will open new avenues in both theoretical and applied biology.

## Introduction

If the inheritance of phenotypic traits was additive, progenies would always exhibit intermediate trait values compared to their respective parents. Genetic nonadditivity is, however, a common result of intraspecific crosses. It has been exploited for decades in agronomy [1–5], although the underlying mechanisms remain a major question for evolutionary genetics and crop science [6,7]. In plants, superior vigour in hybrids compared to the parents, or heterosis, is frequent [8–12], and its molecular bases have been investigated in numerous studies [13–17]. Empirical observations have often led to contrasting conclusions, and none of the proposed genetic mechanisms can fully explain the emergence of heterosis of different traits across all study systems [13,18–23]. Thus, we are still lacking a unifying theoretical corpus that enables us to explain and predict heterosis of fitness-related traits, including biomass, growth rate, and fecundity.

Several genetic hypotheses have been proposed to explain heterosis [7,14,17,24]. According to the ‘dominance hypothesis’, each parent contributes favourable, dominant alleles (generally at many loci) that together complement the deleterious effects of recessive alleles originating from the other parent. The ‘overdominance hypothesis’ postulates the existence of loci in which the heterozygous state (*Aa*) contributes to the superiority of the hybrid compared to both homozygotes (*AA* or *aa*). Pseudo-overdominance corresponds to cases in which dominant, favourable alleles are in linkage with recessive, unfavourable alleles so that the heterozygous combinations appear to behave as overdominant loci. These different mechanisms are not mutually exclusive, and they can all operate at the same time in a given species, which can explain some of the contradictory results observed in different studies. The picture is further complicated by the contributions of epistasis [19,25] and epigenetics [16,26] to heterosis in plants. To tease apart the different hypotheses, quantitative trait locus (QTL) mapping has been carried out in many species [9,13,19,21,25,27]. In general, individual studies differ strongly in their conclusions regarding the underlying genetic mechanisms, apparently depending on the investigated traits, the genetic material used, the mating system, and the experimental constraints related to the number of crosses necessary for robust statistical inference (e.g., diallel mating design) [17,23,24]. In addition, the QTL approach has inherent limitations when it comes to the detection of small-effect loci [28]. It has been shown that fitness-related traits such as growth rate, size, and fruit production are often controlled by a large number of genes, which individually may have very weak effects [29–31], reducing the usefulness of the QTL approach.

Because of the polygenic nature of fitness-related traits, heterosis is expected to be associated with molecular dominance and should positively correlate with genetic distance between parents, at least up to a certain extent [32–35]. Some findings are consistent with this hypothesis [36–38], suggesting that parental genetic distance could be used to quantitatively predict heterosis in plants. However, experimental studies generally employ for practical reasons only a relatively small number of crosses and parental lines [11,35], which makes it difficult to generalize the findings of individual studies. In a recent work, Seymour and colleagues [17] investigated heterosis in a half-diallel cross between 30 genome-sequenced accessions of *A*. *thaliana* collected from diverse Eurasian populations [39]. As expected under the dominance model [32–34], they found a positive correlation between parental genetic distance and heterosis. However, genetic distance between parents only accounted for less than 3% of heterosis among *A*. *thaliana* hybrids, making predictions based on genetic distances alone strongly uncertain.

**Fig 1:**
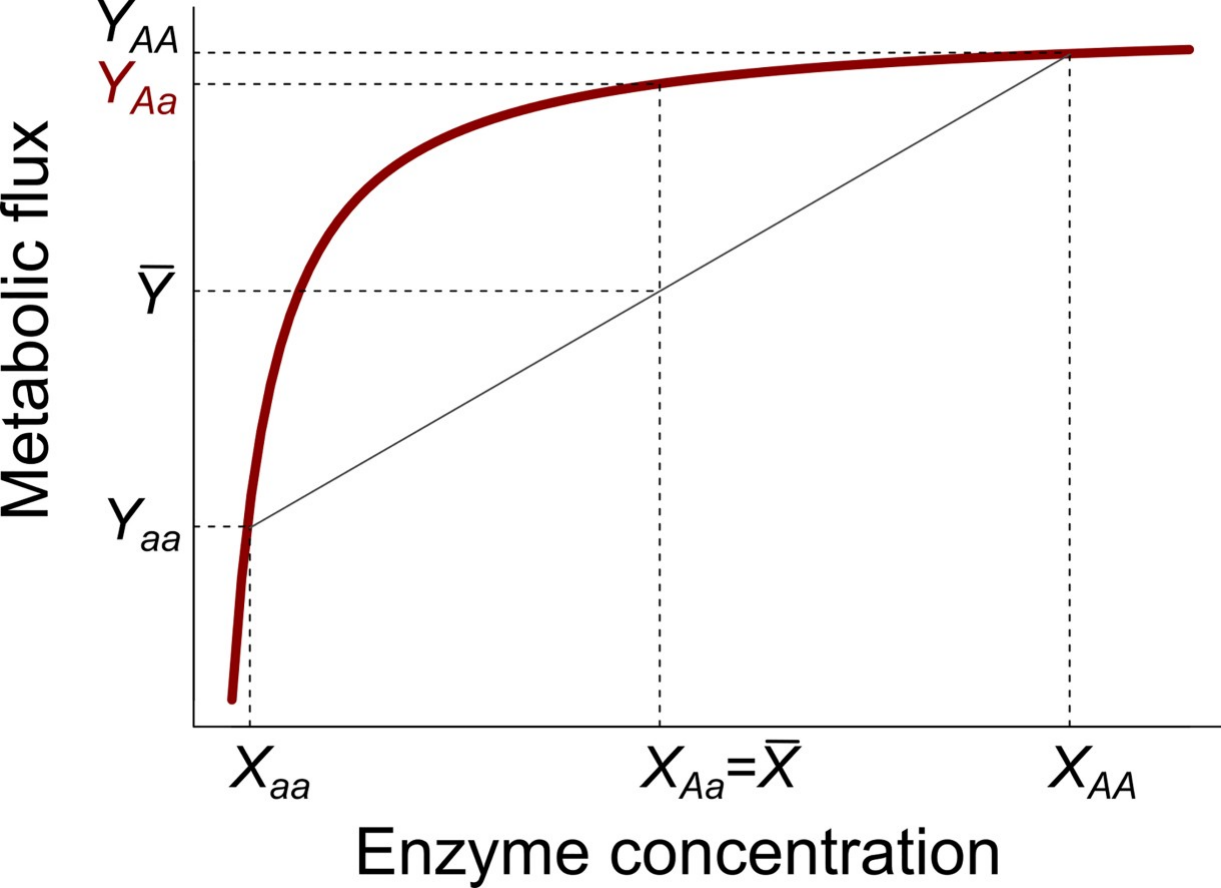
Wright’s model of physiological dominance. The red curve represents the relationship between a metabolic flux (*y*-axis) and the concentration of one enzyme of the system (*x*-axis). The nonlinearity of the relationship results in dominance of allele *A* over allele *a*: the flux of the hybrid *Aa* is higher than the mean parental flux ($\bar{X}$ and $\bar{Y}$ represent mean enzyme concentration value and mean metabolic flux value between parent 1 [*aa*] and parent 2 [*AA*], respectively).

Despite or perhaps because of all of these previous efforts, many continue to consider the physiological bases of heterosis a mystery [40,41]. An alternative to genetics-first studies to understand and predict heterosis is to consider the physiological constraints that determine phenotypic variation across organisms. As early as 1934, Sewall Wright proposed a model of physiological dominance to explain the maintenance of recessive alleles in natural populations [42]. Wright began with the universal relationship that connects the concentration of enzymes to the metabolic flux that results from their activity (Fig 1). Since the relationship between these two traits is concave with a horizontal asymptote (e.g., Michaelis–Menten kinetics [43]), dominance of metabolic flux is expected even if enzyme concentrations are additive and hybrids intermediate between their parents (Fig 1). Modern knowledge of metabolic networks and fluxes was later incorporated into this model by Kacser and Burns in 1981 [44]. However, Wright’s model of dominance has otherwise received little attention in the heterosis literature, presumably because in the 1930s, physiology was synonymous to metabolism, whereas this is not the case in modern scientific language [45]. Recently, Fiévet and colleagues [46,47] evaluated Wright’s model with a focus on the relationship between metabolic flux and enzyme activity in the chain of glycolysis in yeast. They modelled the strength of the curvature of the relationship between enzyme concentration and metabolic flux and validated its role in the emergence of heterosis on glycolysis [46]. This approach has considerable promise for predicting the phenotype of hybrids by considering the nonlinear relationships that often link phenotypic traits with each other. However, these theoretical expectations remain to be tested using more complex traits as well as on multicellular organisms, such as plants, with potentially major perspectives for cultivated species.

**Fig 2:**
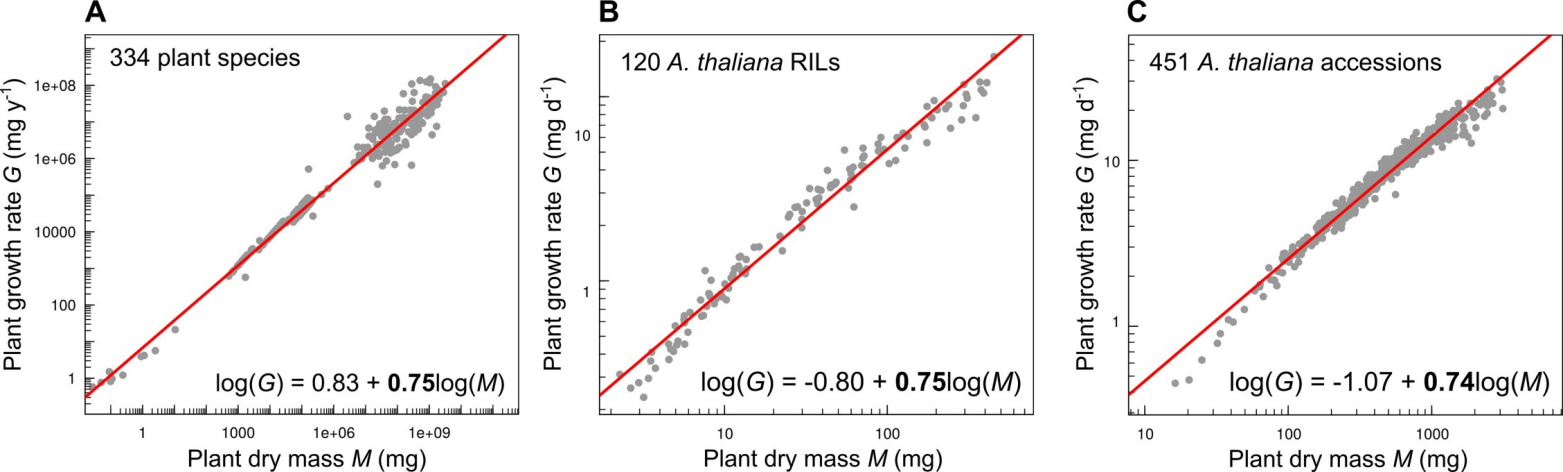
Allometric relationships in plants. (A) Log–log relationship between annual growth rate *G* and plant dry mass *M* across 334 plant species (data from [66]). (B) Log–log relationship between daily growth rate *G* and plant dry mass *M* across 120 RILs of *A*. *thaliana* (data from [60]). (C) Log–log relationship between daily growth rate *G* and plant dry mass *M* across 451 natural accessions of *A*. *thaliana* (data from [31]). Red lines and equations were fitted with SMA regressions. RIL, recombinant inbred line; SMA, standard major axis.

Many phenotypic relationships exhibit nonlinear geometries at different organizational levels. At the cellular level, examples include the relationships between RNA transcripts and protein level [48–50] or the relationship between mitochondrial respiration and cell growth [51]. At the organism level, macroecology studies have demonstrated the existence of nonlinear allometric relationships between the biomass of an organism and its morphology, physiology, and metabolism [52–55] or between reproductive and fitness-related traits [31,53,56]. The metabolic scaling theory (MST) [55] posits that the geometry of the resource distribution network, like vein branching in plants [57,58], constrains allometric relationships to be predictably nonlinear across taxonomic scales [54,59] (Box 1, Fig 2). Consistent with this idea, recent findings demonstrated that the allometric relationship of growth rate is not only similar across species but also across natural accessions of *A*. *thaliana* as well as recombinant inbred lines (RILs) of this species artificially created in laboratory [31,60]. These relationships are analogous to those between metabolic and enzymatic activities described by Wright [42], Kacser and Burns [44], and Fiévet and colleagues [46,47]. For instance, if an additive trait *x* is linked to a trait *y* by an asymptotic relationship, like enzyme concentration is to metabolic flux in Fig 1, hybrids should exhibit mid-parent heterosis (but not best-parent heterosis) for trait *y*. However, Fig 1 represents a simple and unrealistic case in which the metabolic flux depends on a single enzyme. The recent study performed by Fiévet and colleagues [46] demonstrated that in a more realistic situation, the multidimensional relationship between several enzymes and metabolic flux is concave, hump shaped. In such a case, hybrids can exhibit best-parent heterosis, as has been shown for the chain of glycolysis [46].

**Box 1. Allometric relationships: Biological laws of phenotypic variation across taxonomic scales.** Allometric relationships often take the form of a function *Y* = α*M*^β^, in which *Y* is a morphological or physiological trait (e.g., respiration rate, growth rate, leaf area, reproductive biomass) that can be predicted by *M*, the biomass of the organism, a constant α, and the allometric exponent β [52,53,57,58,61,62]. The rationale behind the study of allometric relationships is that organism biomass determines its volume and the number of metabolically active units (cells, mitochondria), which in turn determine the consumption of energy at the organism level, its respiration rate, hydraulic fluxes, and growth rate. Allometric relationships are usually linearized by logarithmic transformation such that log(*Y*) = log(α) + β × log(*M*), especially to compare exponent values β (slope of the log–log relationship) between organisms [63]. Indeed, the allometric exponent of many traits exhibits a value less than one, causing a concavity of the relationship (called ‘strict allometry’ [63]). For instance, the metabolic scaling theory (MST) posits that the distribution network of resources (nutrient, energy) is shaped by universal constraints related to organism size (e.g., branching geometry and space filling) in virtually all organisms [54,55,62]. As a result of these constraints, MST predicts that the allometric exponent β is a multiple of a quarter (e.g., ¼, ¾) for many biological processes, such as respiration rate, total leaf area, photosynthetic rate, and growth rate [52,55,59,64,65]. For instance, plant growth rate scales with a slope of 0.75 to plant biomass across 334 species, spanning more than 10 orders of magnitude in size (data from [66], Fig 2A). MST has generated a vigorous debate among biologists about the supposed universality of 0.75 allometric exponent [67–69]. Recent findings in *A*. *thaliana* demonstrated that plant growth rate also scales with a slope of 0.75 to plant biomass across recombinant inbred lines (RILs; data from [60], Fig 2B) and across natural accessions (data from [31], Fig 2C). This suggests that the allometry of growth rate results from strong biophysical constraints, which operate independently of the taxonomic scale. Since RILs are lines generated by artificial crosses in the laboratory, this also suggests that the allometry of growth rate at 0.75 is prevalent without selection filtering.

Inspired by Wright’s model of physiological dominance for enzyme concentration and metabolic flux [42], we have tested whether allometric relationships can explain the emergence of heterosis in macroscopic traits related to performance, such as growth rate and fruit production. We used the plant *A*. *thaliana*, which has been used for many genetic studies of heterosis [11,17,70–73]. This species is characterized by a high rate of inbreeding, which leads to a high rate of homozygosity in natural accessions, considered as inbreds [74,75]. The complete sequencing of 1,135 natural accessions [76] has provided abundant information on the extent of genetic variation between populations, the genotype–phenotype map of the species, as well as the causes of phenotypic variation in intraspecific hybrids [77–81]. Several studies reported strong diversification of primary and secondary metabolism between accessions [82–86], which points to large genetic variation in enzyme properties between accessions, a key observation for Wright’s model of physiological dominance. Furthermore, allometric relationships that link growth rate and fruit production to plant biomass have been evaluated in this species [31,60,87]. Here, we specifically addressed the following questions: (i) Are there nonlinear allometric relationships that similarly constrain trait variation in natural accessions and hybrids? And (ii) do these nonlinear relationships explain the emergence and extent of heterosis in growth rate and fruit production? To this end, we compared several traits between 451 accessions, representing a wide range of native environments, and 450 crosses derived from crosses among 415 of these accessions. Our results are consistent with many aspects of Wright’s model, suggesting that heterosis emerges intrinsically from nonlinear relationships between traits.

## Results

### Trait variation and heterosis in a wide range of *A*. *thaliana* genotypes

**Fig 3:**
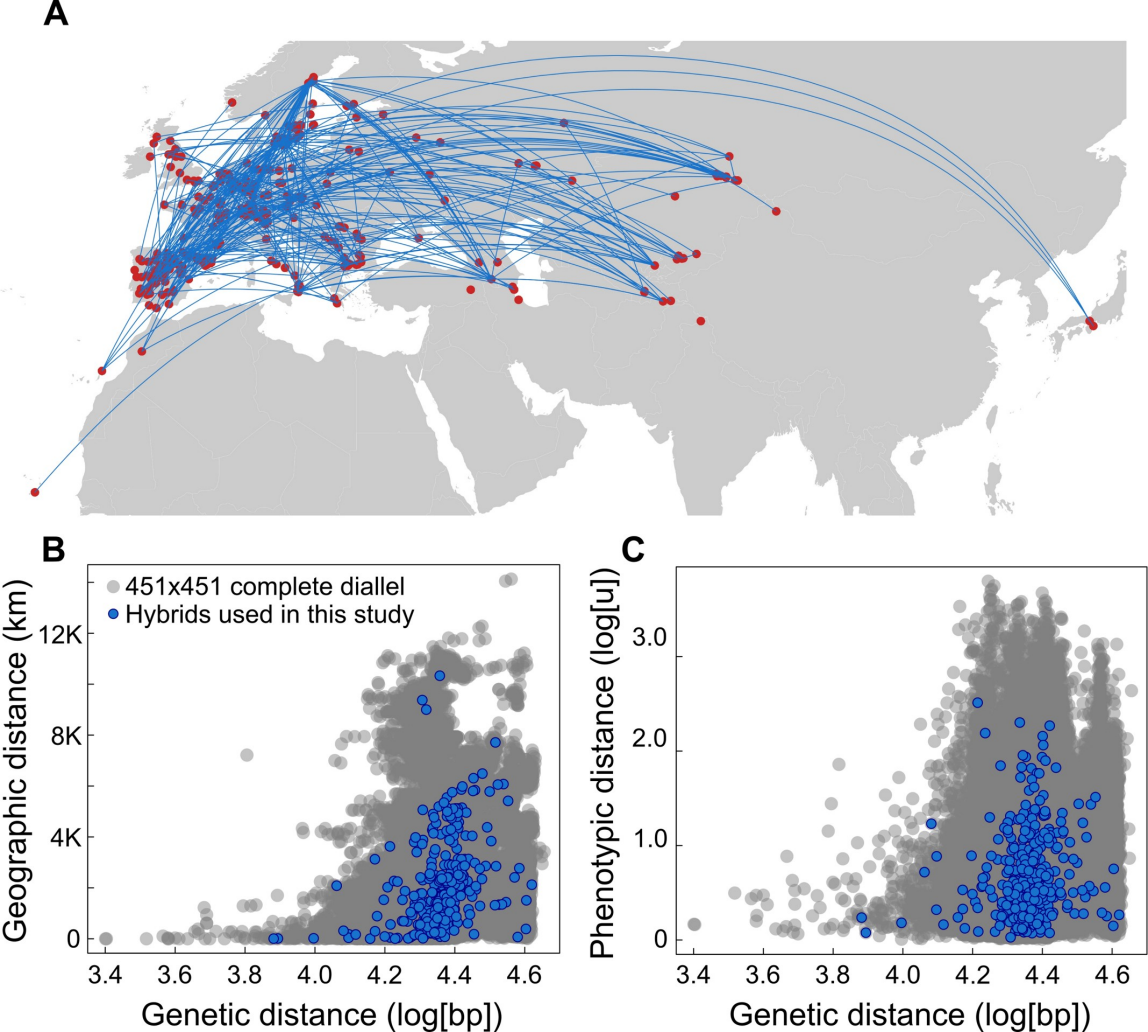
Diversity of genetic crosses used. (A) Origin of 451 natural accessions (red dots) and the 450 hybrids between them (blue connecting lines). (B) Relationship between pairwise genetic distances and pairwise geographic distances. (C) Relationship between pairwise genetic distances and pairwise phenotypic distances (Euclidean distances). Distance–distance relationships are represented between all possible pairs of the 451 accessions (101,475 combinations, grey dots) and the crosses used for the 451 phenotyped hybrids (blue dots).

We generated 450 hybrids by manual crosses between 415 accessions (Fig 3A). We then measured four traits, vegetative dry mass, plant age at reproduction, growth rate, and total fruit number in the 450 hybrids, the 415 inbred parents, plus a further 35 accessions. The parental combinations for the hybrids had been chosen to represent a wide range of genetic, geographic, and phenotypic distances (Fig 3B and 3C).

**Fig 4:**
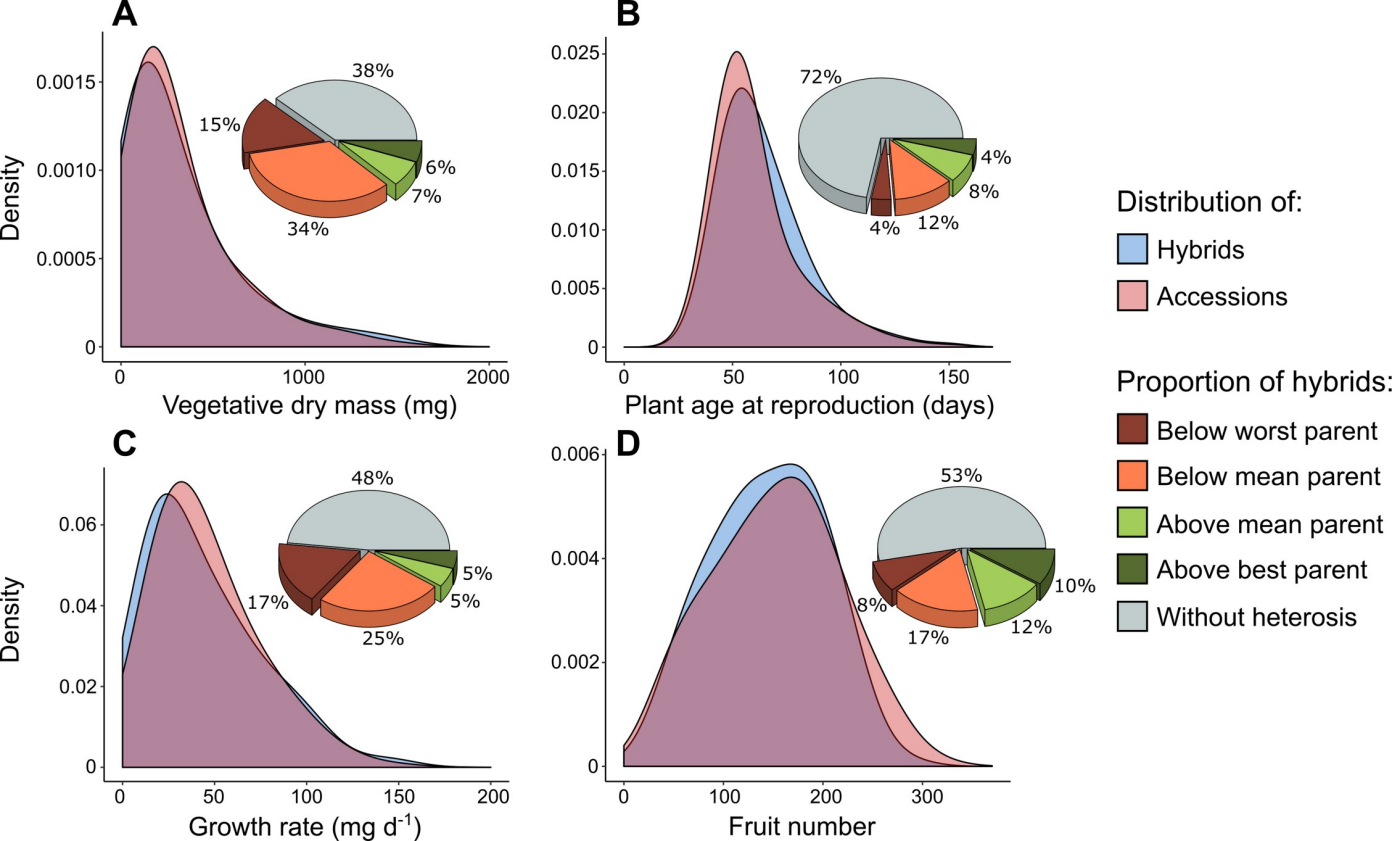
Distributions of traits and proportion of heterosis. (A) Vegetative dry mass *M* in accessions (red) and hybrids (blue). Pie chart indicates proportion of crosses without and with significant heterosis. (B) Plant age at reproduction. (C) Growth rate. (D) Fruit number. Density represents KDE. KDE, kernel density estimation.

Traits were strongly variable between genotypes: vegetative dry mass *M* varied between 1.3 and 2,218 mg, plant age at reproduction between 24 and 183 days, growth rate between 0.04 and 40.4 mg/d^−1^, and fruit number between 5 and 400 (S1 Data). Across the entire data set of hybrids and accessions, a high proportion of the phenotypic variance was explained by genotypic differences (broad-sense heritability): from 80% for growth rate to 94% for vegetative dry mass (S1 Table). We found similar ranges of variation for both accessions and hybrids (Fig 4). For instance, standard deviation of plant age at reproduction was 19.8 days across accessions and 20.1 days across hybrids (S1 Table). Thus, hybrids represent a similar phenotypic space as natural accessions, and they were on average not significantly different from accessions (*P* > 0.01 for all traits, S1 Table).

Despite the similar distribution of trait values for accessions and hybrids, we found significant heterosis for all traits. The emergence of heterosis in our data did not simply reflect a few exceptional hybrids that exhibited trait values outside of the range observed across accessions but rather hybrids with specific combinations of traits within a similar range of trait values. The proportion of hybrids exhibiting significant differences compared to their parents was measured by the discrete categorization of heterosis, based on the comparison of hybrid trait distribution with both mid-parent values and highest (‘best’) or lowest (‘worst’) parent values. For all traits, there was both positive and negative heterosis (Fig 4). However, the extent and direction of heterosis was variable between traits. For instance, 28% of hybrids were (positively or negatively) heterotic for age at reproduction. By contrast, 62% of hybrids were heterotic for vegetative dry mass. In general, there was more negative than positive heterosis, specifically for vegetative dry mass (49% of hybrids).

### Similar nonlinear allometric relationships in accessions and hybrids

**Fig 5:**
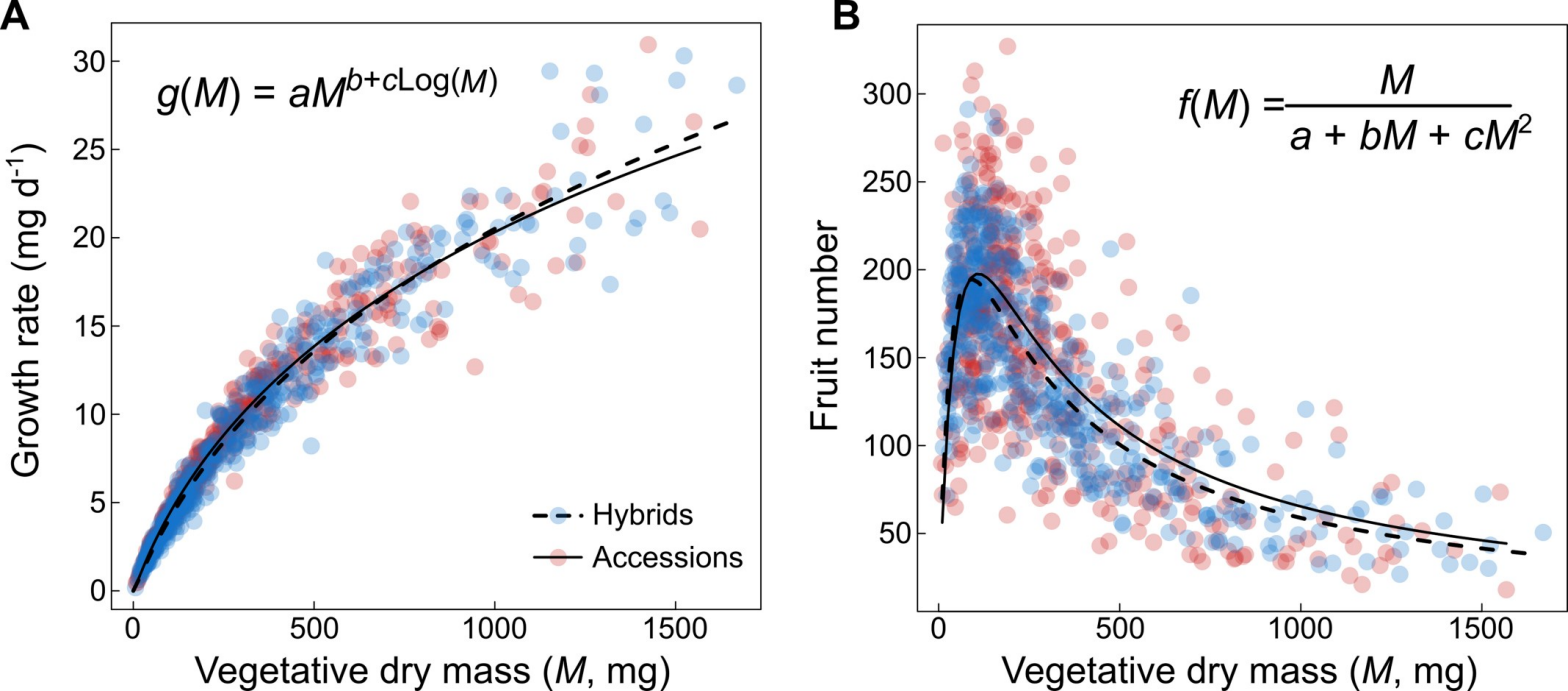
Allometric relationships of growth rate and fruit number. (A) Allometric relationship between growth rate and vegetative plant dry mass *M* fitted with a power-law function with mass-corrected exponent (*g*[*M*]). Equation fitted separately on 451 accessions (red dots, solid line) and 447 hybrids (blue dots, dashed line). (B) Allometric relationship between fruit number and *M*, fitted with an inverse polynomial function (*f*[*M*]). Equation fitted separately on 441 accessions (red dots, solid line) and 449 hybrids (blue dots, dashed line).

We focused on two traits with important evolutionary and agronomic outcomes: growth rate and fruit number. As predicted by the MST [55,58], these two traits exhibited nonlinear allometric relationships with vegetative dry mass (Fig 5). Moreover, in both accessions and hybrids, the allometric relationship of growth rate had an exponent close to ¾ (the slope of the log_10_-transformed relationship was 0.74 for accessions and 0.78 for hybrids, S1 Fig). Nonetheless, standard major axis (SMA) regressions revealed that the slope was significantly different between accessions and hybrids (*P* < 0.01). The slope was not significantly different from ¾ for the accessions (*P* > 0.05) but differed significantly from ¾ for the hybrids (*P* < 0.01). However, and consistent with recent studies of plant allometry [31,60,88], growth rate was more accurately modelled by a power-law function with mass-dependent exponent (*g*[*M*], Table 1) rather than a fixed exponent (Akaike information criterion [ΔAIC] = 53 for the accessions and ΔAIC = 55 for the hybrids). This generated a nonlinear relationship with a concave curvature (Fig 5A), whose coefficients were not significantly different between accessions and hybrids (Table 1). Fruit number exhibited a right-skewed hump-shaped relationship (Fig 5B). We modelled this allometric relationship with an inverse quadratic function (*f*[*M*], Table 1). As for growth rate, accessions and hybrids exhibited similar nonlinear relationships, characterized by coefficients that were not significantly different from each over (Table 1).

**Table 1:** Coefficients of fitted allometric relationships.

		Fitted coefficients (95% CI)		
Equation		*a*	*b*	*c*
*g*(*M*) = *aM*^*b*+*cLog*(*M*)^	Accessions	**0.022** (0.011; 0.043)	**1.483** (1.256; 1.717)	**−0.164** (−0.209; −0.121)
	Hybrids	**0.020** (0.010; 0.039)	**1.456** (1.237; 1.684)	**−0.151** (−0.194; −0.110)
$f(M)=\frac{M}{a+bM+cM^{2}}$	Accessions	**0.155** (0.108; 0.210)	**0.0022** (0.0014; 0.0029)	**1.292 10**^**−5**^ (1.086 10^−5^; 1.524 10^−5^)
	Hybrids	**0.116** (0.094; 0.141)	**0.0025** (0.0021; 0.0030)	**1.444 10**^**−5**^ (1.291 10^−5^; 1.608 10^−5^)

### Relationships between heterosis and genetic, phenotypic, and geographic distances

We first compared what fraction of heterosis can be explained by genetic and phenotypic distances between inbred parents. For all hybrids, we quantified heterosis as the observed phenotypic deviation relative to mid-parent heterosis (MPH) value and best parental heterosis (BPH) value. Pairwise genetic distances were calculated either with all SNPs in the genome or with SNPs in the 1% top-genes associated with the corresponding trait [31]. For the phenotypic distance, we used the absolute difference in vegetative dry mass between parents.

**Fig 6:**
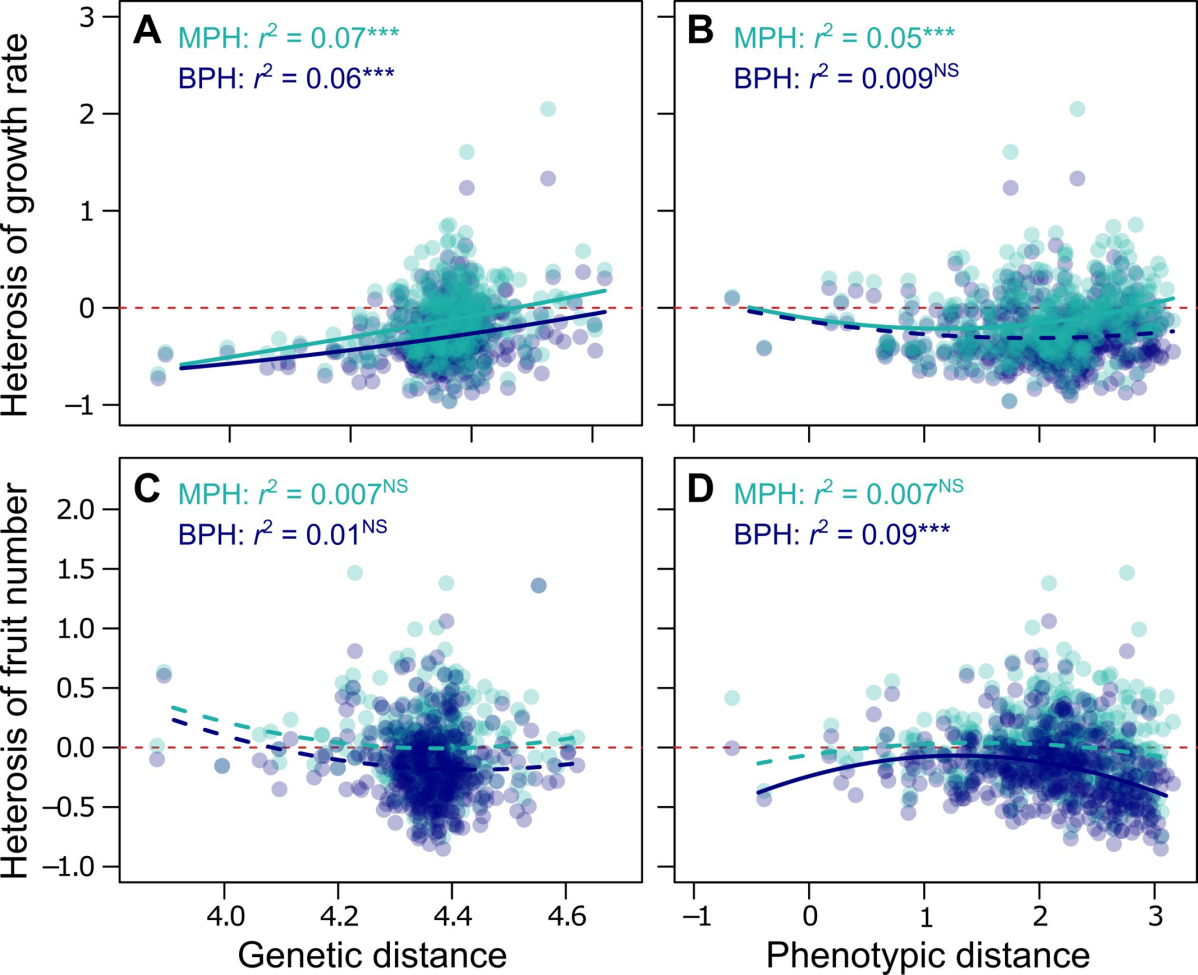
Correlation of heterosis with genetic and phenotypic distances. (A) Correlation between pairwise genetic distances between parental accessions and observed heterosis of growth rate in hybrids (*n* = 368). (B) Correlation between pairwise phenotypic distances between parental accessions (absolute difference in *M*) and observed heterosis of growth rate in hybrids (*n* = 447). (C) Pairwise genetic distances versus heterosis of fruit number (*n* = 368). (D) Pairwise phenotypic distances versus heterosis of fruit number (*n* = 449). MPH (light blue), BPH (dark blue). *r*^2^ are coefficients of correlation (***P < 0.001). Lines were fitted with quadratic models: solid lines when the correlation was significant (*P* < 0.01), dashed lines otherwise. Red dashed lines represent zero axis. BPH, best-parent heterosis; MPH, mid-parent heterosis; NS, nonsignificant.

We fitted quadratic relationships to test for potential nonlinearity. The second-order term was not significant for genetic distance but significant for the relationships between phenotypic distance and MPH of growth rate (Fig 6B) and BPH of fruit number (Fig 6D). This suggests an optimal phenotypic distance for maximization of heterosis, although it was not a good predictor on its own (MPH of growth rate, *r*^2^ = 0.05, Fig 6B; and BPH of fruit number, *r*^2^ = 0.09, Fig 6D). For both traits, the relationships with phenotypic distance did not differ significantly between MPH and BPH (95% confidence intervals [CIs] of all regression coefficients overlapped between MPH and BPH). Genetic distance between parents was positively, and linearly, correlated with heterosis of growth rate (both MPH and BPH *P* < 0.001; Fig 6A) but accounted for less than 10% of heterosis (7% and 6% for MPH and BPH, respectively). By contrast, genetic distance did not correlate with heterosis of fruit number (both P > 0.01; Fig 6C). Using only the 1% top-genes associated with growth rate and fruit number, which are more likely to have a causal role in the measured traits, did not improve correlations (*r*^2^ < 0.10). Even when combined in a multivariate model, genetic and phenotypic distances together explained less than 10% of heterosis of growth rate and fruit number (S2 Table). For comparison, geographic distance between parents explained less than 2% of heterosis of fruit number and less than 0.2% of heterosis of growth rate. In summary, none of the parental distances—be they genetic, phenotypic, or geographic—had much power to explain and predict a significant portion of heterosis.

### Explaining variation in heterosis by phenotypic nonlinearity

**Fig 7:**
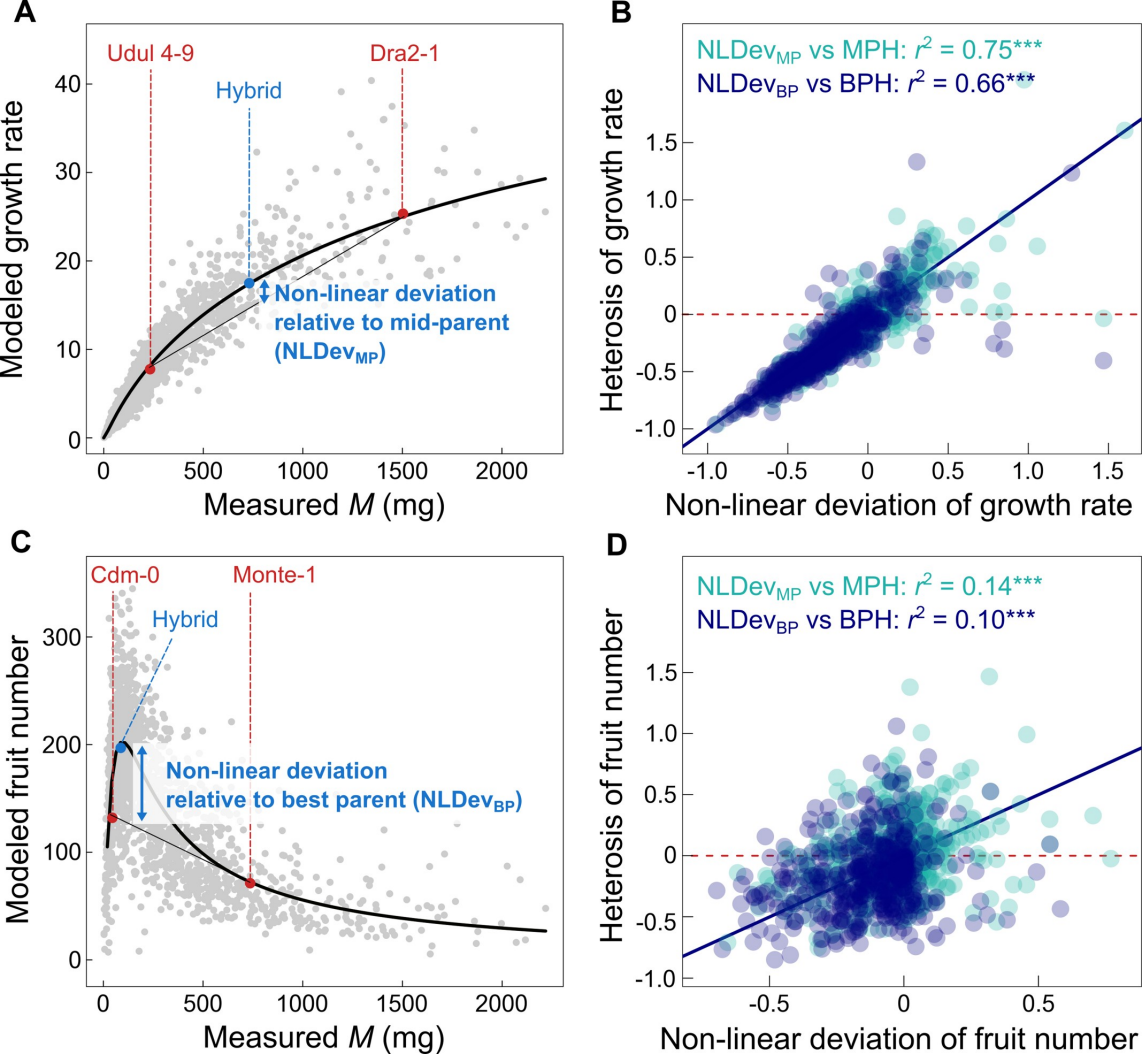
Prediction of heterosis with phenotypic nonlinearity. (A) Allometric relationship of growth rate (*g*[*M*], black solid line) fitted in accessions, with an example of two parental accessions (Udul 4–9 and Dra2-1, in red) and their hybrid (in blue). NLDev (blue arrows) was calculated relative to mid-parent value (MP_pred_): NLDev_MP_ = (*g*[*M*_1 x 2_] − MP_pred_) / MP_pred_ and relative to best-parent value (BP_pred_): NLDev_BP_ = (*g*[*M*_1 x 2_] − BP_pred_) / BP_pred_. (B) Correlation between NLDev and heterosis in all hybrids (*n* = 447), both relative to MPH (light blue) and BPH (dark blue). (C) Allometric relationship of fruit number (*f*[*M*], black solid line) fitted in accessions, with an example of two parental accessions (Cdm-0 and Monte-1, in red) and their hybrid (in blue). NLDev was calculated relative to mid-parent value (NLDev_MP_) and relative to best-parent value (NLDev_BP_). (D) Correlation between NLDev and heterosis in all hybrids (*n* = 447), both relative to MPH (light blue) and BPH (dark blue). Red dashed lines represent zero axes. Black dashed line represents 1:1 line. Grey dots are all hybrid individuals. *r*^2^ are Pearson’s coefficients of correlation (***P < 0.001). BPH, best-parent heterosis; MPH, mid-parent heterosis; NLDev, nonlinear deviation; NS, nonsignificant.

In a second approach, we used the fitted equations (Table 1) to take into account the nonlinearity of allometric relationships and to predict heterosis. Our first goal was to predict growth rate and fruit number with (i) the allometric relationship fitted on parents and (ii) the measurement of vegetative dry mass in hybrids (Fig 7). Predicted growth rate in hybrids strongly correlated with observed growth rate (*r*^2^ = 0.95, S2A Fig). By contrast, predicted fruit number was poorly correlated with observed trait values (*r*^2^ = 0.08, S2B Fig). This is consistent with the larger dispersion of trait values around the fitted curve for fruit number (Fig 5B) compared to growth rate (Fig 5A). We then compared nonlinear deviation of traits predicted in hybrids—relative to the predicted mid-parent value (nonlinear deviation [NLDev_MP_]) and to the predicted best-parent value (NLDev_BP_)—with the observed MPH and BPH (Fig 7). Observed heterosis and predicted NLDev of growth rate were strongly correlated (*r*^2^ = 0.75 and 0.66 for NLDev_MP_ versus MPH and NLDev_BP_ versus BPH, respectively; Fig 7B). Observed heterosis and predicted NLDev of fruit number were also positively correlated, although weaker than for growth rate (*r*^2^ = 0.14 and 0.10 for NLDev_MP_ versus MPH and NLDev_BP_ versus BPH, respectively; Fig 7D). This suggests that allometric relationships allow the prediction of heterosis amplitude and that prediction accuracy depends on the strength of the underlying nonlinear covariation between traits.

## Discussion

Already in 1934, Wright wrote in his seminal paper that ‘dominance has to do with the physiology of the organism and has nothing to do with the mechanism of transmission’ [42]. Eighty years later, the emergence of heterosis is still considered as an enigma, and its physiological bases remain debated. Despite the many dominant, overdominant, and epistatic QTL identified in a plethora of species [23,24,40], none of the genetic models have been formally validated in more than a few cases [13]. Here, we approached the question of heterosis from a physiological angle based on a geometric analysis of trait–trait relationships. Combining the ideas put forth by Wright [42] with principles from metabolic scaling theory (MST) [52,65], we demonstrate that a significant part of heterosis can be explained by the nonlinear relationships that link phenotypic traits with each other.

Wright’s model of physiological dominance [42] was based on the nonlinear relationship that connects two traits at different levels of integration, enzyme concentration, and metabolic fluxes. Consistent with Wright’s model, evolutionary theory suggests that the intensity of inbreeding depression, and hence the potential for heterosis, should increase with the level of phenotypic integration [89]. The questions around the hierarchy of trait integration have been a key objective and enduring challenge for the field of phenotypic integration [90–93]. Following Arnold’s definition [94], a trait is said to be integrated when it results from the action of several underlying traits (and presumably, genes). This leads to a pyramidal view of phenotypic integration, in which gene products (RNA, transcription factors, enzymes) are at the bottom and fitness-related traits (growth rate, fecundity) at the top. In agreement with Arnold’s definition of phenotypic integration, several studies have shown that RNAs and transcription factors can regulate plant growth through their effects on intermediate traits such as plant development, vegetative meristem activities, cell elongation, photosynthetic efficiency, and secondary wall biosynthesis [95–97]. Under these assumptions, biomass is expected to be highly integrated but at a lower level than growth rate and fruit number [98]. Consistent with this view, survival and fertility are more sensitive to inbreeding depression than size, biomass, and gross morphology in animals [99]. In addition, fitness traits show lower heritabilities than morphological traits [100]. Our model supports the idea that integrated traits in plants like growth rate and fruit number exhibit strong heterosis because they result from the multiplicative effects of nonlinear relationships at different organizational levels [101,102], such as abundances of RNA transcriptions and proteins [48–50], mitochondrial respiration and cell growth [51], and allometric laws of biomass allocation [63]. In this study, we focused on modelling the allometric variations of growth rate and fruit number. However, nonlinear relationships at successive organizational levels can have different curvatures and directions, which might either amplify or cancel each other. Moreover, different components of integrated traits could exhibit variable degrees of heterosis depending on how these components are connected to traits at lower levels of integration. For instance, there is an expected trade-off between seed size and seed number [103,104], even though a recent work suggests that seed size can strongly vary without changes in seed number [105]. Thus, since the metabolic pathways of fruit size can differ from the one of fruit number, we could expect that the prediction of heterosis of total seed yield would be complex [106] and that it must rely on the joint analysis of several trait–trait relationships.

The high fraction of negative heterosis observed in our study for plant biomass is consistent with previous findings in *A*. *thaliana* [17,71], although it contrasts with some others [11,73]. It could potentially be explained by a convex relationship with a trait at a lower integration level [107], for instance, a trait related to organism development and phenology. In addition, another explanation to the emergence of negative heterosis is that we used crosses from strongly divergent accessions, which might be incompatible because of deleterious interactions between defence-related genes [108]. Autoimmunity is associated with a reduction of growth and fecundity, and it has been repeatedly observed when crossing distant accessions of *A*. *thaliana* [81]. Moreover, a general repression of the immune system has been observed in hybrids with positive heterosis on biomass [73]. Consistent with a recent suggestion that inferior hybrids are as common within regions as between regions [81], we did not find evidence of a connection between negative heterosis and parental geographic distance (geographic distance was not correlated with MPH and BPH of plant dry mass, *r*^2^ < 0.01).

The genetic bases of integrated traits such as growth rate and fruit production are complex by nature because these traits result from the effects of numerous genes acting on different components of performance [30,101]. Inexpensive high-density genotype information coupled with very detailed phenotyping provides a series of promising avenues for the genomic prediction of heterosis [109]. In this context, the dominance hypothesis implicates, within certain limits, a positive relationship between parental genetic distance and heterosis [32,33]. Results from a range of species, however, do not conform with these expectations [11,17,35,110,111]. One of the reasons could be the often small number of crosses and the relatively small range of genetic distances analysed, with the latter holding true especially in cultivated species [11]. Our results with 450 hybrids representing crosses between diverse *A*. *thaliana* populations pointed to a positive but weak correlation between heterosis and parental genetic distance for growth rate but no such correlation for fruit number. This suggests that a genetic approach alone may not be sufficient to accurately predict heterosis. By contrast, our results indicate that phenotypic nonlinearity determines the emergence of heterosis. We have shown that even if vegetative dry mass exhibited important deviation from additivity, it could still be used to predict heterosis of growth rate and fruit number based on parental allometric relationships. Our model notably predicts that strong heterosis of biomass (e.g., BPH) is always associated with strong heterosis of growth rate. However, strong heterosis of biomass can be associated with negative heterosis of fruit number because fruit number is linked to biomass with a hump-shaped curve. For instance, consider a case in which two parents are to the right of the fitness peak: it is expected that BPH of biomass results in fewer fruits than for either parent. As observed in yeast [112,113], this example illustrates how phenotypic nonlinearity can generate overall positive heterosis despite many single loci that are inherited as underdominant.

The accuracy of heterosis prediction with phenotypic nonlinearity depends on how strongly traits correlate with each other. MST [52,55,57] postulates that body size constrains trait variation in the form of universal mathematical laws (Box 1). According to the mechanistic assumption of MST, biomass integrates the number of metabolically active units (cells, mitochondria). Because the number of metabolically active units is the main driver of physiological and metabolic variation between individuals, biomass is thus expected to predictably determine physiological fluxes, growth rate, and metabolic activity at the organismal level [65,66]. We have found that hybrids exhibit the same allometric relationships as natural inbred accessions. Moreover, the relationship for growth rate is similar to the one observed within and across species as well as in recombinant inbred lines (RILs) that never experienced natural selection [31,60,114] (Fig 2). This reinforces the idea of strong, evolutionary stable and predictable biophysical constraints on the variation of growth rate with biomass. For instance, our method explained up to 75% of heterosis amplitude for growth rate (Fig 7B) while genetic distance at best explained only 7% (Fig 6A). Consistent with the present study, Flint-Garcia and colleagues [10] with maize, Seymour and colleagues [17] as well as Palacio-Lopez and colleagues [35] with *A*. *thaliana*, all found very weak correlation between heterosis and genetic distance between parents (*r*^2^ < 0.10).

The prediction of heterosis using the nonlinearity of the allometric relationship was lower for fruit number (14% for MPH), which can be attributed to the higher variation of this trait around the fitted curve. Fruit number is difficult to measure accurately, so a possible explanation to this low prediction compared to growth rate is that our protocol to estimate fruit number was prone to a certain degree of uncertainty (e.g., measurement errors due to time of sampling, image analysis). Alternatively, we can suppose that fruit number depends on much more traits than just biomass and that it is more plastic than growth rate to small environmental variations. For instance, source/sink competition between vegetative biomass and reproductive output, visible here by the decrease of fruit number after a certain size threshold (Fig 5B), is expected to vary in response to the abundance of resources. Previous studies have shown that the nonlinearity of relationships between traits and genetic markers that explain heterosis in maize [115] and jack pine [107] was more pronounced under stressful conditions. This suggests that environmental factors could play an important role in the manifestation of heterosis by increasing the curvature of trait relationships [116–118]. Since allometric relationships are expected to vary with the environment [87,119]—in particular the relationship between plant biomass and fruit production—an open question that needs to be addressed in the future is how genotype-by-environment interactions impact the prediction of heterosis through their effects on trait covariation.

Predictions of heterosis can reach relatively high levels in specific biparental populations in which dominant or overdominant QTL have been mapped (e.g., [13,27]), but such QTL cannot be generalized to other populations with different allelic composition and genetic determinism of traits. Several authors have proposed powerful methods based on genomic predictions to predict hybrid performance, such as de Abreu e Lima and colleagues [120], who predicted up to 41% of hybrid performance in maize; Zhao and colleagues [121], who predicted up to 89% of hybrid performance in wheat; and Werner and colleagues [122], who predicted up to 82% of hybrid performance in oilseed rape. However, these studies predicted hybrid performance, i.e., hybrid trait value, and not heterosis per se (i.e., the deviation of hybrid trait value to the parental mean value or parental best value). For crop breeding, it is hybrid performance that is the fundamental unit of financial care, but it is possible that a good prediction of hybrid performance fails to accurately predict the emergence and amplitude of heterosis. For instance, our study suggests that high-performance hybrids are not necessarily those that exhibit the strongest amplitude of heterosis (0.31 < *r*^2^ < 0.47; S3 Fig). Inversely, if a hybrid has high heterosis but is not in the top for hybrid performance, then it is not useful in a crop setting. Our study thus calls for an assessment of whether predictive models of hybrid performance are good predictive models of heterosis.

From a theoretical point of view, the predominance of outcrossing and the maintenance of recessive alleles among organisms have been suggested to be directly linked to nonadditive inheritance and superior performance of hybrids [123]. Wright’s initial model of physiological dominance was proposed as a response to Fisher’s idea that ‘gene modifiers of dominance’ must exist and be selected to maximize the fitness of the heterozygote [124]. Wright [42], and later Kacser and Burns [44], claimed that modifiers are not necessary because nonlinearity is an intrinsic characteristic of metabolic networks. The same argument holds true for complex traits such as growth rate and fruit number. The assumption of modifiers of dominance is based on the unrealistic expectation of an intermediate phenotype in the heterozygote, while phenotypic relationships are essentially nonlinear [46,63,125]. A next step in getting to the physiological root of heterosis will be the integration of other, more low-level traits, especially gene expression and metabolite levels. We expect that this will improve the characterization of nonlinear relationships in multidimensional phenotypic space and ultimately shed light on the physiological mechanisms at the origin of nonadditive inheritance.

## Conclusions

The development of a predictive approach for heterosis is a long-term goal of modern biology, especially in the applied framework of varietal selection in crops [126]. Our study has shown that trait variation is similarly constrained in accessions and hybrids of *A*. *thaliana*, which highlights the power of a geometric approach of trait–trait relationships for predicting heterosis related to two major components of plant productivity and yield. It opens promising avenues for cultivated species with a perspective of targeting optimal crosses based on allometric relationships in parental lines. However, several differences between crops and *A*. *thaliana* might hamper the prediction of heterosis based on allometric relationships. For instance, the genetic architecture of metabolism and performance-related traits could differ in crops compared to *A*. *thaliana*. Strong selection for high-yielding lines in crops may have reduced genetic variability in modern varieties and may have modified to a certain degree the relationship between genetic and phenotypic distance. Deleterious recessive alleles may have been purged in cultivated species as a result of domestication and human selection. Unfortunately, most plant species that have been evaluated for inbreeding depression and heterosis are cultivated species, and we lack a proper comparison of wild and cultivated species regarding these processes. In addition, traits other than biomass (harvesting date, plant height) could be relevant for prediction of hybrid performance in crops, especially in field conditions. For instance, plant height might be more related to grain yield than biomass, as suggested by the quadratic relationship between these traits in wheat [127]. With respect to understanding the emergence of heterosis of complex traits, the analysis of the relationships between traits and metabolomic profiles is promising. For instance, correlations between performance-related traits and the metabolome have been reported in *A*. *thaliana* [85], rice hybrids [128], and maize hybrids [6]. It is now time to test the phenotypic approach to heterosis in crops, for which the study of allometric relationships is a nascent research front [129–131].

## Material and methods

### Plant material

For inbred genotypes, 451 natural accessions of *A*. *thaliana* were phenotyped in 2014 at the Max Planck Institute for Developmental Biology in Tübingen (MPI-Tübingen), Germany (*n* = 2, Exp 1, S1 Data). They are the same individuals as those used for the analysis of the natural variation in growth rate and plant allometry [31,132]. These accessions were chosen to represent a wide geographic area: a majority of them were derived from Russia and European countries, with two additional accessions from Japan and a few from North Africa. To generate the 450 hybrids phenotyped in a second experiment in 2015 at MPI-Tübingen (*n* = 4, Exp 2, S1 Data), 415 accessions were used as parental lines and randomly crossed. Among the parental accessions, 342 were used as female parent and 318 as male parent. Genetic data were available for 407 accessions among the 451 total and 369 accessions among the 415 parental, as they had been genome sequenced as part of the 1001 Genomes project (http://1001genomes.org/) [76]. Among the 415 parental accessions, 134 (32%) were used in a single cross, 166 (40%) were used in two crosses, 63 (15%) in three crosses, and 52 (13%) in at least four crosses. To overcome potential maternal effects, the same mother plants grown in the greenhouse in 2013 provided the seeds for both the inbred accessions (by self-fertilization) and the hybrids (by manual cross) (S4A Fig).

Data for the 334 plant species shown in Fig 2A were obtained from the study by Niklas and Enquist [66], with unicellular algae removed. The data consist of annual growth rate estimates across aquatic and terrestrial metaphytes, including herbaceous dicots as well as arborescent monocots, dicots, and conifers. For terrestrial metaphytes, traits were measured on even-age monospecific stands grown under horticulturally controlled field [66]. Most of the data were originally compiled by Cannell [133] based on the primary literature published up to mid-1981 for approximately 1,200 forest stands from 46 countries.

### Growth conditions

We designed a hydroponic system in which plants were cultivated on inorganic solid media (rockwool), and all nutrients were provided through the watering solution. Circular pots (Pöppelmann, Lohne, Germany) of 4.6 cm (diameter) x 5 cm (depth) were filled with 3.6 cm x 3.6 cm x 4 cm depth rockwool cubes (Grodan cubes, Rockwool International, Denmark). Pots were covered with a black foam disk with a 5–10 mm central circular opening. Seeds were sown in individual pots, randomly distributed in trays of 30 pots each (S4B Fig).

Before sowing, all seeds were surface-sterilized with 100% ethanol and frozen overnight at −80°C to kill any insect eggs. The rockwool cubes were placed in 75% strength nutrient solution, as described in ref. [134] in order to achieve full humidification and fertilization. After sowing on the surface of the rockwool cubes, trays with 30 pots each were incubated for 2 days in the dark at 4°C for stratification. Trays were transferred for 6 days to 23°C (8-h day length) for germination. After 6 days, when most seedlings had two cotyledons, trays were transferred to 4°C (8 h light) for 41 days of vernalization in order to reduce the range of flowering times among accessions. During germination and vernalization, all trays were watered once a week with a 75% strength nutrient solution. After vernalization, when true leaves had emerged on most individuals, plants were thinned to one individual per pot, and trays were moved to the Raspberry Pi Automated Plant Analysis (RAPA) facility [132] (S4B Fig), set to 16°C, air humidity at 65%, and 12-h day length, with a PPFD of 125 to 175 μmol m^−2^ s^−1^ provided by a 1:1 mixture of Cool White and Gro-Lux Wide Spectrum fluorescent lights (Luxline plus F36W/840, Sylvania, Germany). Trays were randomly positioned in the room and watered every 1 to 3 days with 100% strength nutrient solution.

### Trait measurement

Plants were grown and phenotyped using rigorously the same protocol, following methodologies previously published for accessions [31,132]. Plants were harvested at the end of the life cycle when the first fruits were senescing. Flower production ceased at this stage, so only a few fruits were expected to be newly produced when the first fruits were drying [132]. Consistently, when we left the plants under well-watered conditions after the senescence of the first fruits, we could not detect the formation of new stems, and fruit yield was only marginally increased.

At harvesting, rosettes were separated from roots and reproductive parts, dried at 65°C for 3 days, and weighed. Plant age at reproduction (d) was measured as the duration between the appearance of the two first leaves after vernalization and the end of the life cycle [132]. Growth rate (mg/d^−1^) was calculated as the ratio of final rosette dry mass over plant age at reproduction. Inflorescences were photographed with a high-resolution, 16.6 megapixel SLR camera (Canon EOS-1, Canon Inc., Japan) and analysed with ImageJ [135] to estimate the number of fruits through 2D image skeletonization, following published protocols [31,132].

The RAPA system was used for daily imaging using 192 microcameras (OmniVision OV5647), which simultaneously acquired 6 daily top-view 5-megapixel images for each tray during the first 25 days after vernalization. We used a published method to estimate plant dry mass during ontogeny from top-view rosette pictures [31,132]. From fitted sigmoid growth curves on all individuals, we calculated inflection point (d) at which daily growth was maximal and started to decrease. We used rosette dry mass (mg) at the inflection point as measurement of vegetative dry mass *M* (mg). In total, trait values for plant age at reproduction, growth rate, and vegetative dry mass were available on 451 accessions and 447 hybrids and fruit number on 441 accessions and 449 hybrids (S1 Data) [136].

To correct for potential biases between the two experiments performed, 16 accessions phenotyped in Exp 1 were also included in Exp 2. Among all traits measured, only plant age at reproduction exhibited a significant difference between the two experiments (*P* = 0.03). We thus corrected trait values in Exp 2 with the following equation: age_corrected_ at reproduction = −37 + 1.8 × age_observed_ at reproduction.

### Measurement of heterosis

First, hybrid phenotypic classes were categorized by comparing trait distribution in hybrids to mean and best (or worst) parental values. Trait distributions for hybrids were obtained from a bootstrap approach with 1,000 permutations. Each F1 trait distribution was compared to the mean parental value using a two-sided *t* test (ɑ = 0.05) as well as to the worst and best parent values using a one-sided *t* test (ɑ = 0.05). To determine the significance of heterosis categories (below or above mean/best/worst parental values), *P* values were adjusted for multiple testing by a Bonferroni correction.

Secondly, two metrics commonly used in the literature to quantify heterosis [17] were calculated in the present study for all traits *Y*, i.e., growth rate and fruit number:

1. MPH, the deviation of observed hybrid value scaled to observed mean parental value:
  $$MPH=Y_{1\;x\;2}-mean\left(Y_{1},Y_{2}\right)/mean\left(Y_{1},Y_{2}\right)$$
2. BPH, the deviation of observed hybrid value scaled to observed best parental value:
  $$BPH=Y_{1\;x\;2}-max\left(Y_{1},Y_{2}\right)/max\left(Y_{1},Y_{2}\right)$$

### Allometric relationships and measurement of phenotypic nonlinearity

The allometric equations were fitted by the nonlinear least-squares method using the *nls* function in R [137]. Following MST model [31,52,60,88], we chose a power-law equation for growth rate with a mass-corrected allometric exponent (*g*[*M*] in Table 1). The corrected exponent corresponds to the derivative of the quadratic function obtained after logarithmic transformation of the allometric relationship [31,60,88]. In our data, biomass correction of the exponent improved the fitting of the allometric relationship of growth rate (ΔAIC = 53 and 55 for accessions and hybrids, respectively). For the allometric relationship of fruit number, we compared the fitting between a Ricker function (*y* = *axe*^−*bx*^) and inverse polynomial function. The inverse polynomial equation (*f*[*M*] in Table 1) was retained based on AIC (ΔAIC = 12 and 22 for accessions and hybrids, respectively). Confidence intervals (95% CIs) of the fitted coefficients were estimated with the *confint* function in R.

We used the allometric equations fitted on the accessions to predict the phenotype of the hybrids from vegetative dry mass *M*. We first estimated growth rate of both parents and hybrids (*g*[*M*_1_], *g*[*M*_2_], *g*[*M*_1 x 2_], with *M*_1_ and *M*_2_ corresponding to vegetative dry mass of worst and best parent, respectively, and *M*_1 x 2_ to hybrid dry mass). We then predicted mean parental value of growth rate as MP_pred_ = (*g*[*M*_1_] + *g*[*M*_2_])/2 and best parental value as BP_pred_ = max(*g*[*M*_1_], *g*[*M*_2_]). Finally, we predicted the phenotypic nonlinearity as

1. the nonlinear deviation of predicted hybrid value scaled to predicted mean parental value (Fig 7A):
  $$NLDev_{MP}=\left(g\left[M_{1\;x\;2}\right]-MP_{pred}\right)/MP_{pred}$$
2. and the nonlinear deviation of predicted hybrid value scaled to predicted best parental value (Fig 7C):
  $$NLDev_{BP}=\left(g\left[M_{1\;x\;2}\right]-BP_{pred}\right)/BP_{pred}.$$

Both NLDev_MP_ and NLDev_BP_ were calculated for growth rate, and we performed similarly for estimating the nonlinearity of fruit number (Fig 7C).

### Genetic, geographic and phenotypic distances

Out of the 451 natural accessions phenotyped here, 407 accessions have been genome sequenced (http://1001genomes.org/) [76]. Using vcftools [138], the 12,883,854 single-nucleotide polymorphisms (SNPs) were first filtered to retain those in which minor allele frequency was above 5%, with a genotyping rate above 85% across all accessions. This resulted in 391,016 SNPs. We used PLINK v1.9 [139] to estimate pairwise genetic distances as the number of alleles that differed between pairs of accessions (*—distance* function) after log_10_-transformation. We also measured genetic distances using the 1% SNPs with the strongest (positive and negative) effects on growth rate or fruit number from a previously published study [31]. SNPs effects on each trait were estimated using a polygenic GWA model implemented in GEMMA (‘Bayesian Sparse Linear Mixed Model’ [BSLMM]) [140].

Pairwise geographic distances between accessions were estimated from their longitude–latitude coordinates [76] and the *distm* function of the geosphere package in R. For the calculation of pairwise phenotypic distances, we first used Euclidean distance among all traits measured in accessions (vegetative dry mass, age at reproduction, growth rate, fruit number; used for Fig 1). We also used absolute difference in vegetative dry mass between parents for comparing the contribution of genetic and phenotypic distances to heterosis in Fig 6.

### Statistical analyses

Pearson correlation and regression coefficients were calculated using R. The effect of the experiment on trait values was tested on the 16 accessions common to Exp 1 and Exp 2 using two-way ANOVA, with genotype and experiment as interacting factors. To determine heterosis categories (Fig 4), F1 trait distributions were estimated with a nonparametric bootstrap approach across 1,000 permutations using the *boot*.*ci* function of the *simpleboot* R package and compared to mean, worst, or best parental values for each F1 with a one- or two-sided *t* test. *P* values were corrected for multiple testing by a Bonferroni approach. To calculate the proportion of phenotypic variance associated with genotypic (*G*) effects (a measure of broad-sense heritability, *H*^2^ = var[*G*] / [var(*G*) + residuals]), we fitted a mixed model (*lmer* function in R) as *Y* = Genotype + residuals, in which *Y* is trait value and Genotype is used as random factor. Statistical analyses were conducted in R v3.2.3 [137]. Allometric relationships on log_10_ scale (S1 Fig) were fitted with SMA regressions, using the package *smatr* [141] in R. Smooth distributions of trait values (Fig 4) were obtained with kernel density estimation (KDE), a nonparametric way to estimate the probability density function, using the *geom_density* function of the *ggplot2* package in R, with a bandwidth of 150 mg for vegetative dry mass, 9 days for plant age at reproduction, 3 mg/d^−1^ for growth rate, and 25 for fruit number.

## Supporting information

S1 Data Phenotypic data used in this study. (XLSX)

S1 Table Summary statistics of traits measured. (XLSX)

S2 Table Correlation of heterosis with genetic and phenotypic distances. (XLSX)

S1 Fig Allometric relationships between growth rate and vegetative dry mass on a log scale. Slopes measured from SMA regressions after log_10_-transformation of trait values in the 451 accessions (red dots and line) and 447 hybrids (blue dots and line). Slopes after log_10_-transformation represent the scaling exponent without transformation. SMA, standard major axis.
(TIF)

S2 Fig Correlation between predicted and observed hybrid phenotype. (A) Hybrid growth rate (mg/d^−1^) predicted by phenotypic nonlinearity (brown dots) and genetic additivity (mean growth rate between parents, yellow dots) and compared to observed hybrid value (*n* = 447). (B) Hybrid fruit number predicted by phenotypic nonlinearity (brown dots) and genetic additivity (mean fruit number between parents, yellow dots) and compared to observed hybrid value (*n* = 449). *r*^2^ are Pearson’s coefficients of correlation (***P < 0.001). Dashed line represents 1:1 line. NS, nonsignificant.
(TIF)

S3 Fig Relationships between hybrid performance and heterosis. (A) Relationship between hybrid growth rate and MPH of growth rate (*n* = 447). (B) Relationship between hybrid growth rate and BPH of growth rate (*n* = 447). (C) Relationship between hybrid growth rate and MPH of growth rate (*n* = 449). (D) Relationship between hybrid growth rate and BPH of growth rate (*n* = 449). *r*^2^ are Pearson’s coefficients of correlation. BPH, best-parent heterosis; MPH, mid-parent heterosis.
(TIF)

S4 Fig Crossing and phenotyping conditions. (A) Seed production experiment performed in 2013 at MPI-Tübingen (Germany). After manual crossing or self-fertilization, mother flowers were isolated in small paper bags until fruit ripening. Seeds for accessions and hybrids used in this study came from the same mother plants. (B) RAPA growth chamber at MPI-Tübingen with trays of accessions phenotyped during Exp 1 in 2014. MPI-Tübingen, Max Planck Institute for Developmental Biology in Tübingen; RAPA, Raspberry Pi Automated Plant Analysis.
(TIF)

## Abbreviation

AIC: Akaike information criterion
BPH: best-parent heterosis
KDE: kernel density estimation
MPH: mid-parent heterosis
MST: metabolic scaling theory
NLDev: nonlinear deviation
SMA: standard major axis
QTL: quantitative trait locus
RIL: recombinant inbred line
