# Supplementary figures and images for: Nonlinear phenotypic variation uncovers the emergence of heterosis in Arabidopsis thaliana
Source: PLoS Biol. 2019 Apr 24;17(4):e3000214. doi: 10.1371/journal.pbio.3000214 (PMC6481775; doi:10.1371/journal.pbio.3000214)

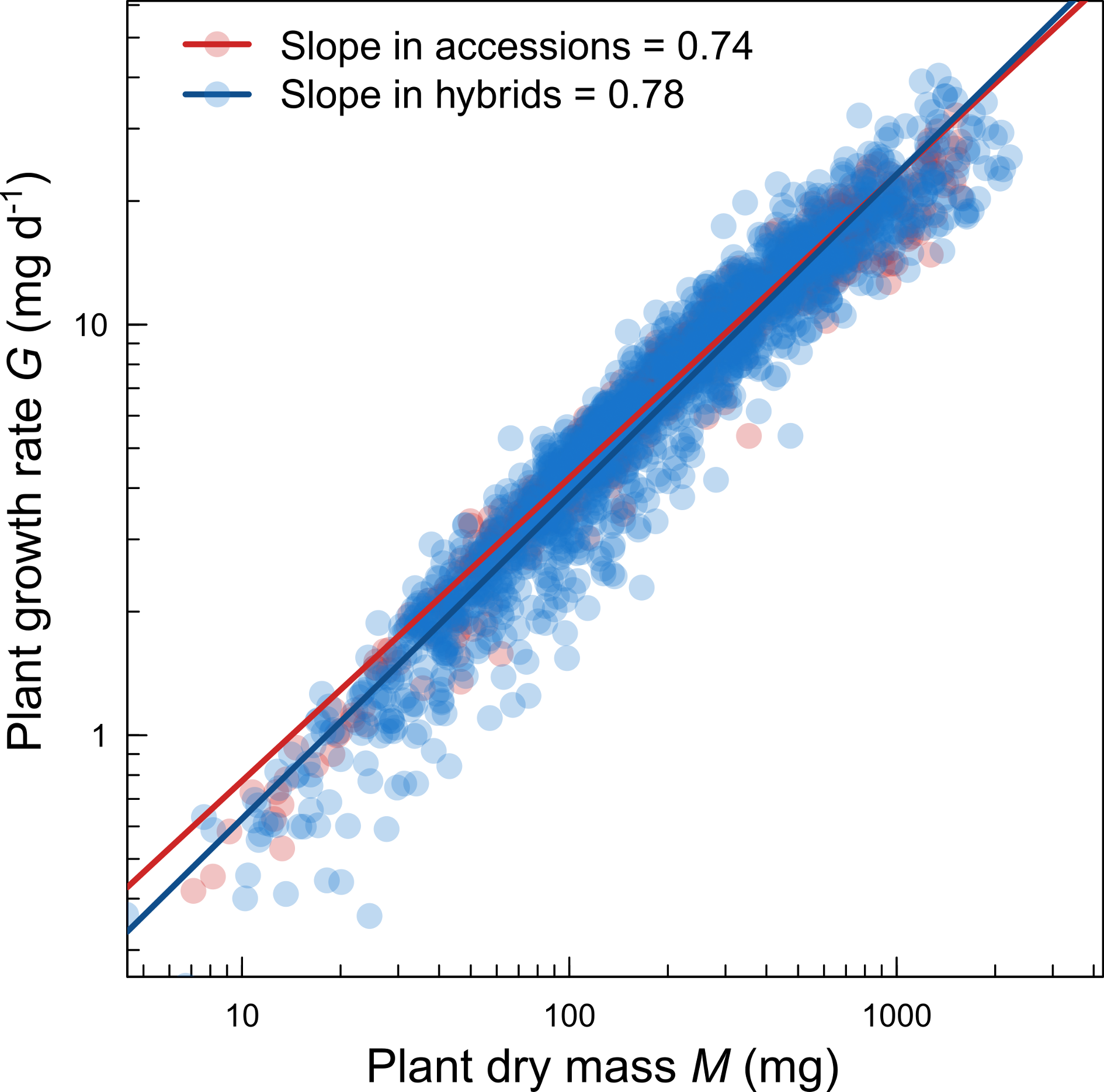

Supplement: S1 Fig — Slopes measured from SMA regressions after log10-transformation of trait values in the 451 accessions (red dots and line) and 447 hybrids (blue dots and line). Slopes after log10-transformation represent the scaling exponent without transformation. SMA, standard major axis. (TIF) [file pbio.3000214.s004.tif]

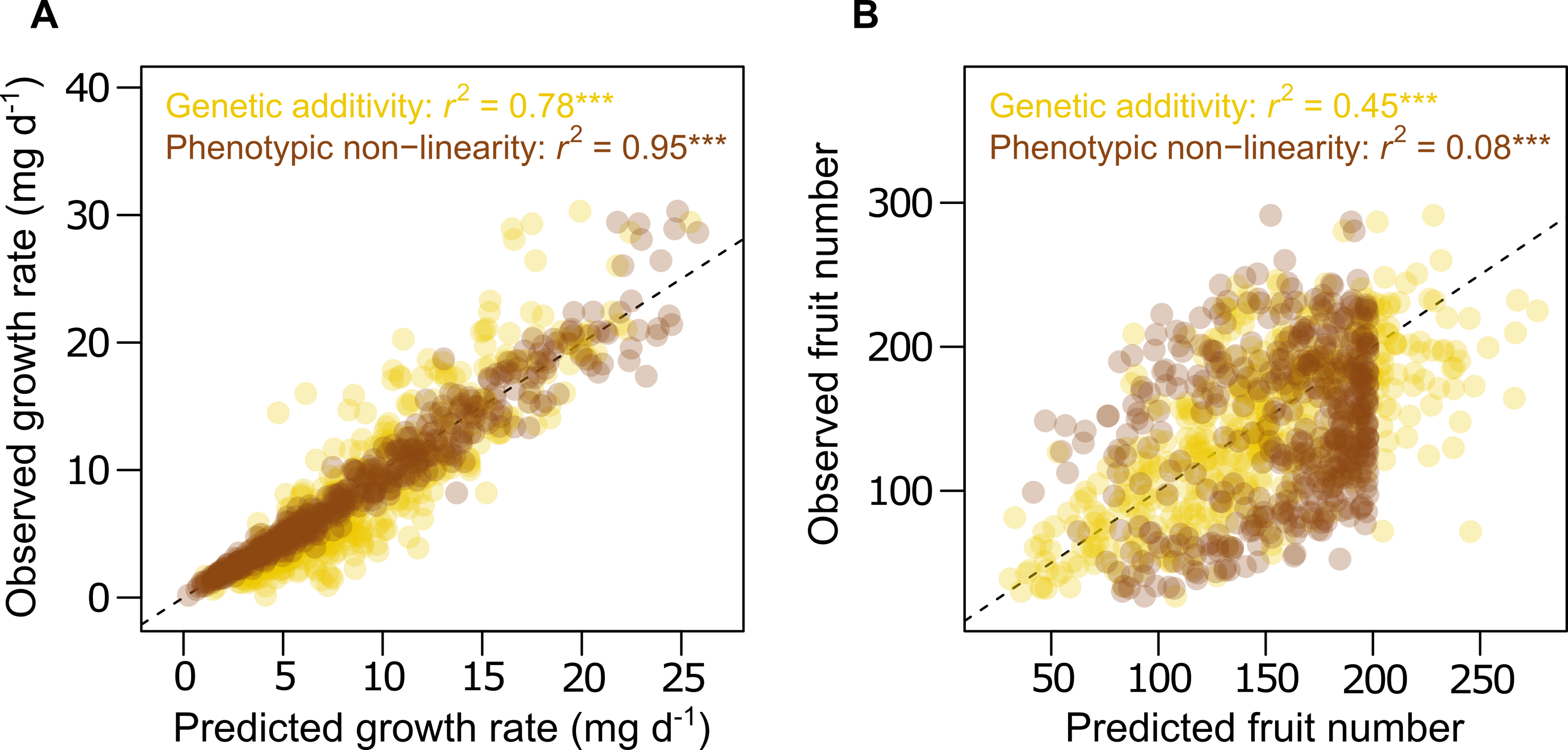

Supplement: S2 Fig — (A) Hybrid growth rate (mg/d−1) predicted by phenotypic nonlinearity (brown dots) and genetic additivity (mean growth rate between parents, yellow dots) and compared to observed hybrid value (n = 447). (B) Hybrid fruit number predicted by phenotypic nonlinearity (brown dots) and genetic additivity (mean fruit number between parents, yellow dots) and compared to observed hybrid value (n = 449). r2 are Pearson’s coefficients of correlation (***P < 0.001). Dashed line represents 1:1 line. NS, nonsignificant. (TIF) [file pbio.3000214.s005.tif]

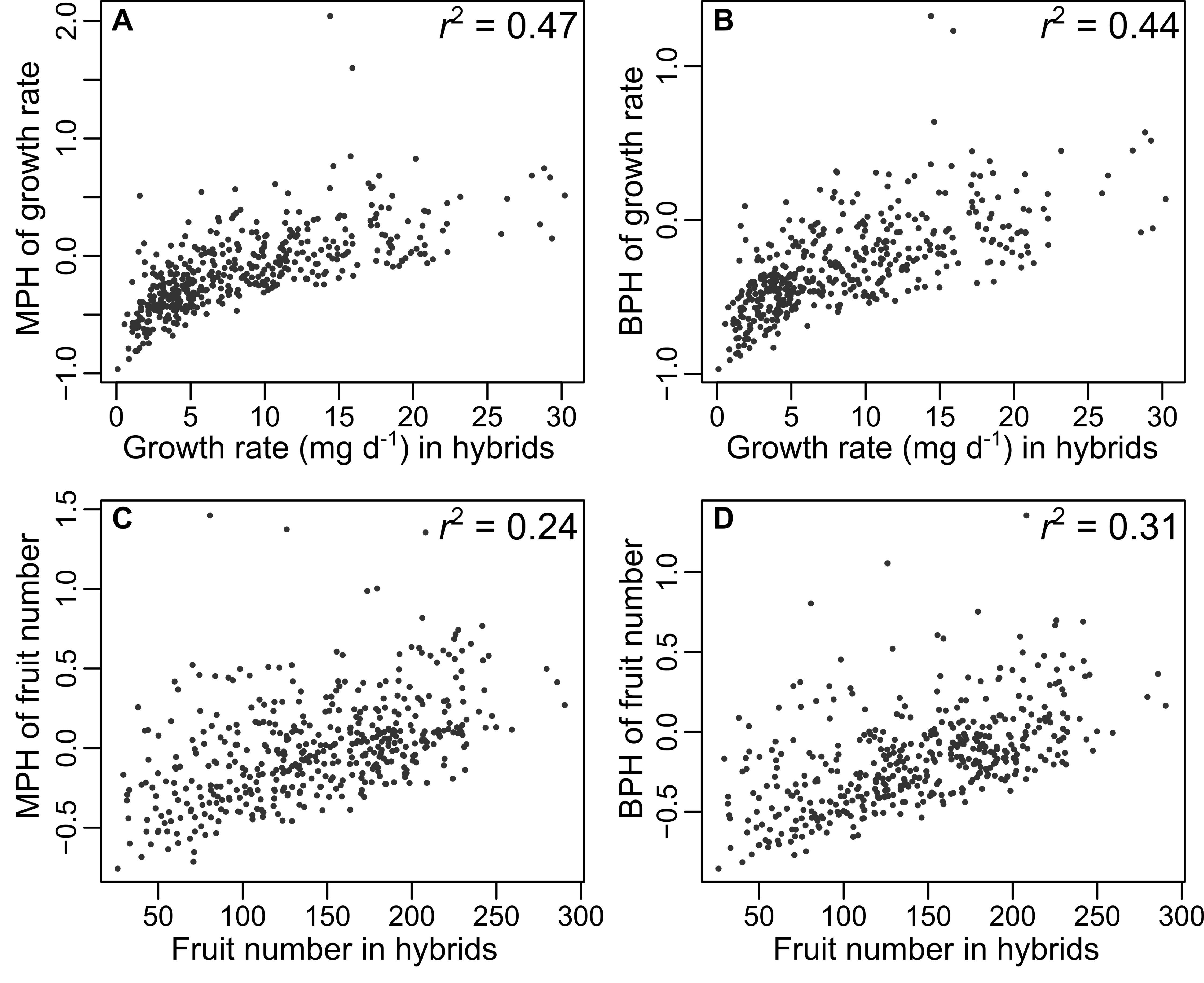

Supplement: S3 Fig — (A) Relationship between hybrid growth rate and MPH of growth rate (n = 447). (B) Relationship between hybrid growth rate and BPH of growth rate (n = 447). (C) Relationship between hybrid growth rate and MPH of growth rate (n = 449). (D) Relationship between hybrid growth rate and BPH of growth rate (n = 449). r2 are Pearson’s coefficients of correlation. BPH, best-parent heterosis; MPH, mid-parent heterosis. (TIF) [file pbio.3000214.s006.tif]

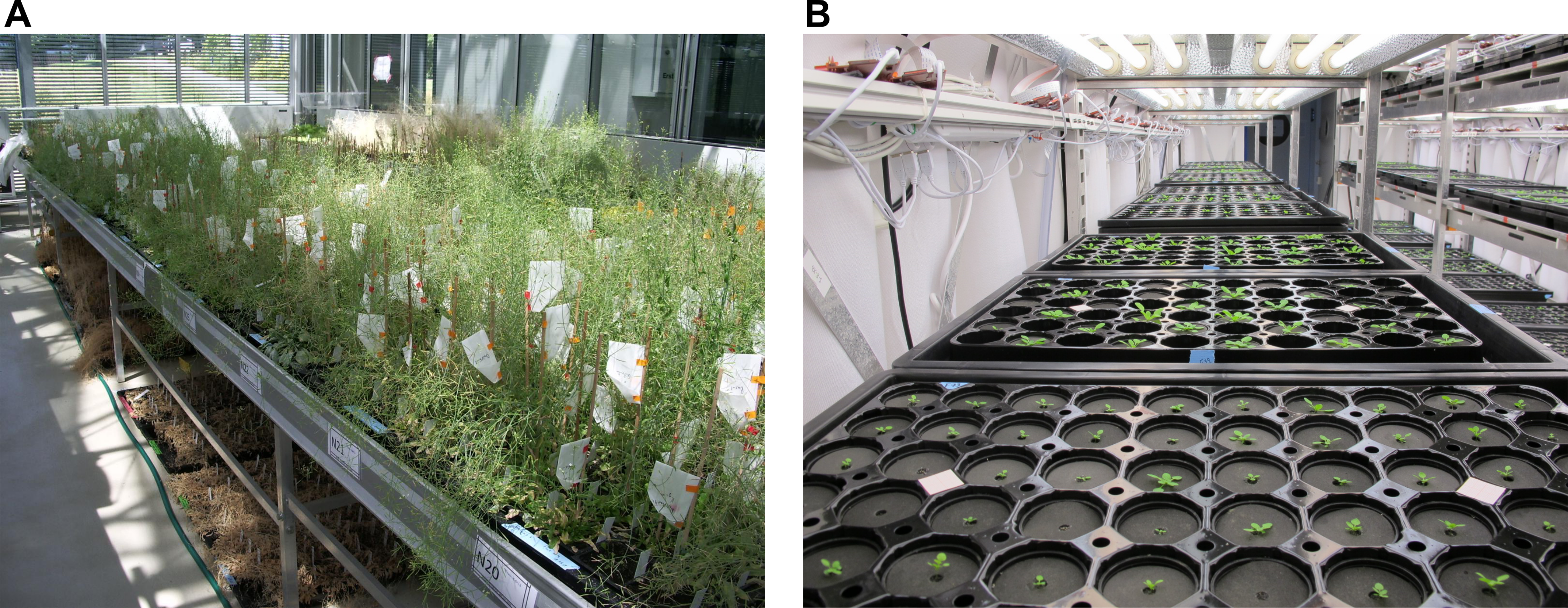

Supplement: S4 Fig — (A) Seed production experiment performed in 2013 at MPI-Tübingen (Germany). After manual crossing or self-fertilization, mother flowers were isolated in small paper bags until fruit ripening. Seeds for accessions and hybrids used in this study came from the same mother plants. (B) RAPA growth chamber at MPI-Tübingen with trays of accessions phenotyped during Exp 1 in 2014. MPI-Tübingen, Max Planck Institute for Developmental Biology in Tübingen; RAPA, Raspberry Pi Automated Plant Analysis. (TIF) [file pbio.3000214.s007.tif]
